# Obesity-related metabolic and endocrine disorders diagnosed during postoperative follow-up of slipped capital femoral epiphysis

**DOI:** 10.1080/17453674.2018.1445167

**Published:** 2018-03-09

**Authors:** Hanifi Ucpunar, Ismet Yalkin Camurcu, Serda Duman, Esra Ucpunar, Hakan Sofu, Avni Ilhan Bayhan

**Affiliations:** 1 Erzincan University Faculty of Medicine, Department of Orthopaedics and Traumatology; 2 Diyarbakir Selahaddin Eyyubi State Hospital, Department of Orthopaedics and Traumatology; 3 Erzincan University Faculty of Health Sciences, Department of Public Health; 4 Baltalimani Bone and Joint Diseases Education and Research Hospital, Department of Pediatric Orthopaedics, Turkey

## Abstract

**Background and purpose:**

Patients with slipped capital femoral epiphysis (SCFE) are phenotypically overweight or obese and may therefore require clinical follow-up of obesity-related disorders. We evaluated obesity-related disorders such as dyslipidemia, type 2 diabetes mellitus (DM), and vitamin-D deficiency during the postoperative period in patients with SCFE.

**Patients and methods:**

51 patients who were operated and followed-up for SCFE and 62 healthy adolescents without SCFE (control group) were included in this retrospective study. Patients’ BMI, serum lipid profile (total cholesterol, LDL-C, HDL-C, triglyceride), fasting blood glucose, HbA1c, and serum vitamin D levels were evaluated.

**Results:**

At the time of surgery, 45 patients in the SCFE group were overweight or obese (BMI >25). At the latest follow-up, 42 patients in the SCFE group and 53 patients in the control group were overweight/obese. Abnormal serum lipid profile and ratio of total dyslipidemia were similar between the groups. 8 patients had abnormal HbA1c levels in the SCFE group and mean HbA1c levels were significantly higher in the SCFE group (p = 0.03). All patients and controls had low levels of vitamin D.

**Interpretation:**

Although serum lipid profile and vitamin D levels were detected as similar in SCFE and control groups, the potential risk of type 2 DM identified via abnormal HbA1c levels was significantly higher in patients with SCFE. We recommend that patients diagnosed with SCFE should be considered as potential candidates for type 2 DM; thus follow-up after surgical treatment should include not only orthopedic outcomes but also evaluation of future risk for DM.

The incidence of slipped capital femoral epiphysis (SCFE) varies with demographic factors such as age, sex, and ethnicity (Loder et al. 2008). However, the exact etiological factors and the leading pathologic mechanism has not yet been clarified. Constitutional femoral retroversion, high BMI, increased vertical slope of the proximal femoral physeal line, and endocrine pathologies have been reported as potential predisposing factors for the pathogenesis of the disorder (Weiner 1996, Witbreuk et al. 2013).

Although the majority of the adolescents diagnosed with SCFE may not have hormonal, metabolic, or chronic diseases they have been reported as phenotypically obese and fast growing (Witbreuk et al. 2013). Novais and Millis (2012) reported that the incidence of SCFE was correlated with increased BMI. Due to this relationship of the disease with obesity, the patients may require clinical follow-up of obesity-related systemic disorders (Murray and Wilson 2008). Additionally, clinical management of these patients may become more complicated because it is still unclear whether the hormonal abnormalities are the reason for or the result of SCFE.

The purpose of this study was to evaluate obesity-related metabolic and endocrine disorders such as dyslipidemia frequency, the prevalence of type 2 diabetes mellitus, and serum vitamin D levels during the clinical follow-up in patients who underwent surgical treatment for SCFE in comparison with overweight/obese healthy adolescents without SCFE.

## Patients and methods

### Study population

Clinical data of the patients were retrospectively evaluated. The SCFE group consisted of patients who were surgically treated between 2008 and 2014 with the diagnosis of unilateral or bilateral idiopathic SCFE. Control individuals were recruited from the adolescents admitted to our outpatient clinic. The control group consisted of mainly overweight or obese (BMI >25) otherwise healthy adolescents without SCFE. Patients and controls with an established diagnosis of any specific systemic, metabolic, or endocrine disorder at the time of surgery or who were under medical treatment which has potential negative effects on glucose, lipid, and vitamin D metabolism were excluded from the study. 51 patients with SCFE and 62 patients without SCFE were eligible for the study (Figure).

**Figure:**
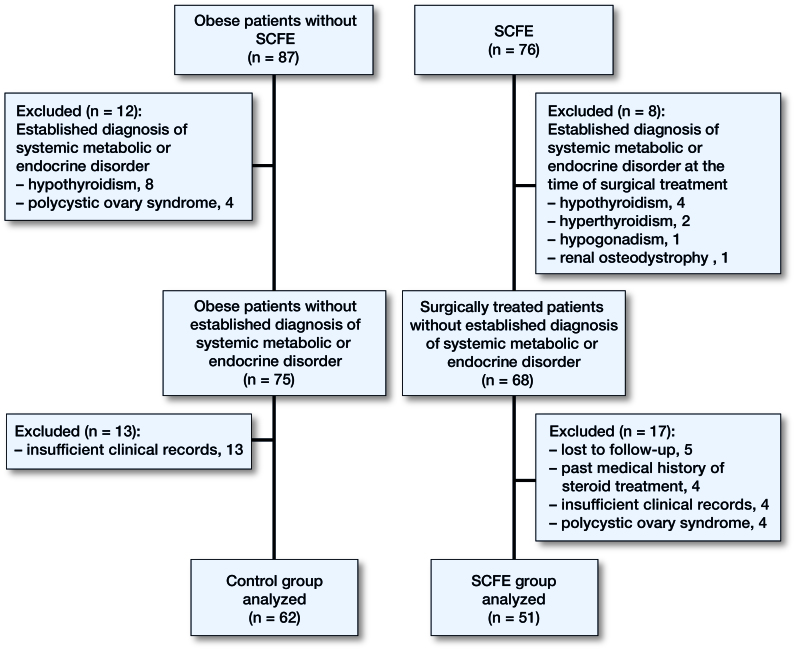
Flow chart demonstrating excluded patients.

### Clinical data collection

The clinical data of the 2 groups were obtained from our clinical database by analyzing laboratory test results and clinical consultation notes of the pediatrics department. The BMI of all patients at the first control as well as the latest follow-up visit were recorded. BMI values were classified using the percentile chart as underweight when <5%, normal weight between 5% and 85%, overweight between 85% and 95%, and obese >95% (Ogden et al. 2002).

Blood samples for evaluation of the serum levels of various parameters were obtained following a 12-hour fasting period without any previous change in the routine diet of the patients.

Serum levels of total cholesterol (TC), low-density lipoprotein cholesterol (LDL-C), high-density lipoprotein cholesterol (HDL-C), and triglyceride (TG) were determined by enzymatic colorimetric method to evaluate the lipid profile. The results were classified as acceptable, borderline high, and high for TC, LDL-C, and TG whereas acceptable, borderline low, and low for HDL-C (NCEP 1992). The patients with high serum levels on any of the measured TC, LDL-C, or TG as well as those with low or borderline low HDL-C were identified as dyslipidemia. Fasting blood glucose (FBG) was determined by enzymatic colorimetric method and hemoglobin A1c (HbA1c) using the immunoturbidimetric method. Fasting blood glucose >125 mg/dL was defined as impaired fasting glucose (IFG). According to an HbA1c test, the patients with a result of ≤5.7% were classified as normal glucose tolerance (NGT), between 5.7% and 6.4% as at risk for diabetes mellitus (DM), and >6.5% as obvious type 2 DM (Nowicka et al. 2011). The serum vitamin D (25-OH-cholecalciferol) level was determined using the electrochemiluminescence immunoassay (ECLIA) method. The results were classified as deficient (< 20 ng/mL), insufficient (21–29 ng/mL), and sufficient (> 30 ng/mL) (Holick 2009). We also recorded serum levels of free tri-iodothyronine (F-T3), free thyroxine (F-T4), thyroid stimulating hormone (TSH, creatinine (kinetic colorimetric test, Jaffé compensated), urea (Urease method), alanine transaminase (ALT)-aspartate aminotransferase (AST) (kinetic method, IFFC without pyrphosp.), which was determined using the electrochemiluminescence immunoassay (ECLIA) method. All biochemical tests were analyzed with an Auto-Analyzer (Roche/Hitachi Cobas c501; Roche Diagnostics USA, Indianapolis, IN, USA).

### Statistics

Statistical analysis was performed using the MedCalc Statistical Software version 12.7.7 (MedCalc Software bvba, Ostend, Belgium; http://www.medcalc.org; 2013). The normality of continuous variables was investigated by the Shapiro–Wilk test. Descriptive statistics were presented using mean (SD) for continuous variables. Non-parametric statistical methods were used for values with skewed distribution. For comparison of 2 non-normally distributed groups the Mann–Whitney U-test was used. The chi-square test was used for categorical variables and expressed as observation counts (and percentages). Fisher’s exact test was used instead of a chi-square test when there was at least 1 expected value less than 5. Multivariate binary logistic regression analysis was used to determine the relationship between HbA1c and independent variables such as SCFE, overweight or obesity, blood lipid profile, fasting blood glucose, and vitamin-D levels. Statistical signiﬁcance was accepted when a 2-sided p-value was <0.05.

### Ethics, funding, and potential conflicts of interest

This study was performed after receiving approval from the institutional ethical review board (33216249-604.01.02-E.16361). Informed consent was obtained from all patients and from individuals in the healthy control group. No funding was received for this study. All authors declare that they have no conflict of interest regarding the submission and publication of this manuscript.

## Results

Table 1 summarizes demographic data of the patients and controls. 45 of the 51 patients in the SCFE group were either overweight or obese at the time of surgery. At latest follow-up, the number of the overweight or obese patients in the SCFE group had decreased to 42. In the control group 53/62 individuals were overweight or obese. There was no statistically significant difference between the 2 groups regarding TC, LDL-C, HDL-C, and TG levels (Table 2). Total dyslipidemia frequency in the SCFE group was 68% and in the control group it was 59% with no statistically significant difference between the groups. According to serum HbA1c levels, 8 patients were diagnosed as either at risk of DM or with obvious type 2 DM, and 2 of the SCFE patients were diagnosed as IFG according to the FBG test (Table 3). In the control group, 2 adolescents were classified as at risk of DM according to HbAc1 levels. The frequency of elevated HbA1c levels and higher mean serum HbA1c were statistically significantly different in SCFE group when compared with controls (Table 3). Multivariate binary logistic regression analysis showed a significant relationship of HbA1c only for the SCFE group (Table 4). Serum vitamin D measurements showed that none of the individuals in either group had a sufficient level of 25-OH-cholecalciferol, and this was similar between the groups (Table 3). F-T3, F-T4, TSH, creatinine, urea, ALT, and AST were measured within normal reference ranges in both the SCFE and control group.

**Table 1.** Demographic data

		SCFE group (n = 51)	Healthy group	
Data	At time of surgery	Last control	(n = 62)	p-value
Female/male^a^	4 / 47		5 / 57	1^c^
Age (years)^b^	13.4 (11–16)	16.6 (16–23)	16.9 (15–21)	0.9^d^
CI	[13.0–13.7]	[16.1–17.1]	[16.1–16.9]	
Follow-up (years)^b^	–	3.1 (2–7)	–	
BMI percentile^b^	91 (65–99)	89 (59–99)	90 (65–98)	0.9^d^
CI	[89–94]	[87–92]	[87–92]	
BMI percentile^a^				
Obese	16	13	15	
Overweight	29	29	38	0.6^e^
Normal weight	6	9	9	

a Number of patients.

b Values are mean, (range), and [95% confidence interval].

c p-values (2-sided) according chi-square/Fisher’s exact test and

d p-values (2-sided) according to Student’s t-test. p-values indicate comparison of last control values in SCFE group and values in healthy group.

e p-value that expresses comparison of frequencies of obese or overweight individuals in the SCFE group and control group.

**Table 2.** Comparison of last-follow up serum lipid profile and serum 25-OH vitamin D levels of the patients versus control group

	SCFE group (n = 51)		Control group (n = 62)		p-value^a^	p-value^b^
Lipid profile (mg/dL)						
Total cholesterol^c^	36 / 8 / 7	160 (33) [150–169]	47 / 8 / 7	158 (30) [151–166]	0.9	0.5
LDL-C^c^	30 / 6 / 15	122 (96) [95–149]	41 / 14 / 7	112 (89) [89–135]	0.2	0.7
TG^c^	18 / 5 / 28	167 (142) [127–207]	29 / 25 / 8	149 (134) [114–184]	0.3	0.2
HDL-C^d^	14 / 13 / 24	45 (11) [42–48]	10 / 17 / 35	46 (10) [43–49]	0.4	0.7
Vit-D (25-OH)						
(ng/mL)^e^	0 / 39 / 12	17 (8) [15–19]	0 / 46 / 16	17 (8) [15–19]	0.7	0.6

a p-values indicate the comparison of the sum of borderline-high and high frequencies of total cholesterol, LDL-C, TG, the sum of borderline-low and low frequencies of HDL-C and insufficient frequencies of Vit-D of the 2 groups.

b Adjusted by sex and age.

c Values are number of patients with acceptable/borderline high/high levels, mean serum level (SD), and [95% CI]

d Values are number of patients with low/borderline low/acceptable levels, mean serum level (SD), and [95% CI]

e Values are number of patients with sufficient/deficient/insufficient levels, mean serum level (SD), and [95% CI]

**Table 3.** Comparison of last follow-up HbA1c and fasting blood glucose levels

	SCFE group (n = 51)	Control group (n = 62)	p-value	
HbA1c				
Serum level (mg/dL)^a^	5.5 (0.3) [5.4–5.6]	5.3 (0.2) [5.3–5.4]	< 0.01^c^	0.04^d^
Normal glucose tolerance^b^	43	60	0.04^e^	
At risk for diabetes mellitus^b^	7	2		
Type 2 diabetes mellitus^b^	1	0		
Fasting blood glucose				
Serum level (mg/dL)^a^	99 (14) [95–103]	99 (SD) [95–102]	0.7^c^	
Normal fasting glucose^b^	49	62	NS	
Impaired fasting glucose^b^	2	0		

NS = not studied

a Values are mean (standard deviation), and [confidence interval]

b Values are number of patients.

c p-value indicates the comparison of the means of serum levels of HbA1c and fasting blood glucose of two groups according to Mann–Whitney U-test.

d Adjusted by sex and age.

e p-value indicates the comparison of the sum of at risk for diabetes mellitus and type 2 diabetes mellitus frequencies of two groups according to chi-square/Fisher’s exact (F) test.

**Table 4.** Multivariate logistic regression analyses of potential high HbA1c level related to confounding variables

	OR (Exp(B))	95% CI	p-value
SCFE	6.0	1.1–32	0.03^a^
Blood glucose	1.3	0.9–1.1	0.4
BMI percentile	1.2	0.9–1.5	0.7
Age	1.2	0.6–2.4	0.5
LDL-C	0.9	0.9–1.0	0.4
Total cholesterol	1.0	0.9–1.0	0.7
Triglycerides	1.0	0.9–1.0	0.2
Vitamin D	0.9	0.8–1.0	0.3

OR = odds ratio

a HbA1c levels were found to be related only to the SCFE group. Other variables, especially weight, which has the potential to raise HbA1c levels, were irrelevant. The confidence interval is rather wide because the numbers of abnormal HbA1c results, particularly for non-SCFE cases, are very small.

## Discussion

The clinical management of patients with SCFE has evolved to a multidisciplinary treatment approach because of their accompanying endocrine and metabolic disorders as well as obesity-related health conditions. Impaired mobility status, secondary skeletal deformities like tibia vara, and SCFE during the pubertal age, as well as systemic disorders such as glucose intolerance, type 2 DM, hyperlipidemia, non-alcoholic fatty liver, cholelithiasis, and primary hypertension, are reported with increased prevalence in overweight or obese individuals (Norman et al. 2005, Taylor et al. 2006, Newmark and Anhalt 2007, Torun et al. 2013). More than 80% of the patients with a diagnosis of SCFE were reported as obese individuals (Manoff et al. 2005). Nasreddine et al. (2013) evaluated 173 cases of SCFE and noted that the ratio of obese patients was 80% preoperatively, which decreased to 78% during a 3-year follow-up period. Aversano et al. (2016) demonstrated an association between BMI-for-age and risk for bilateral SCFE at presentation as well as overall incidence of developing bilateral SCFE in obese children. They concluded that by defining the at-risk population through BMI-for-age, physicians could develop early strategies for therapeutic weight loss, which may reduce the incidence of SCFE. Furthermore, prophylactic pinning of the contralateral hip in unilateral SCFE cases with a BMI-for-age >95% according to percentile chart was also mentioned as one of the mostly discussed controversies in the SCFE literature (Manoff et al. 205). In our patients treated surgically for SCFE, four-fifths of the patients were either overweight or obese preoperatively and this ratio was reduced only slightly postoperatively. Obviously, persistently high BMI ratios demonstrated the underestimated clinical relevance of weight-control programs as well as the ignored importance of obesity and obesity-related disorders in patients surgically treated for SCFE.

Obesity-related metabolic and endocrine disorders have negative effects on physeal development during the fast growing phase of the pre-pubertal and pubertal ages (Witbreuk et al. 2013). Dyslipidemia was reported in between 40% and 45% of obese children of school age in Germany and Turkey (Korsten-Reck et al. 2008, Cizmecioğlu et al. 2008, Elmaogullari et al. 2015). It was emphasized that atherosclerosis-related systemic disorders during adulthood could begin in childhood and adolescence (Berenson et al. 2016). Elmaogullari et al. (2015) mentioned that the risk of dyslipidemia increased in correlation with age and BMI. The ratio of dyslipidemia was similar between the groups in our study, and additionally no significant differences were observed between groups regarding TC, LDL-C, HDL-C, and TG. Our results are consistent with previous studies evaluating the lipid profile of overweight or obese children in similar age groups (Korsten-Reck et al. 2008, Cizmecioğlu et al. 2008, Elmaogullari et al. 2015, Berenson et al. 2016). However, no specific study exists evaluating the lipid profile in children with SCFE and comparing results with obese individuals in the literature. The data that we acquired in this study are insufficient to explain whether dyslipidemia was an etiologic factor for the slippage or simply the result of obesity-related metabolic syndrome.

It is well known that most obese individuals have accompanying blood glucose abnormalities as well as type 2 DM (Pinhas-Hamiel et al. 1996). Bowen et al. (2009) observed 3 separate obesity-related phenotypes in adolescents with no overlap of disease at initial presentation among SCFE, adolescent tibia vara, and type 2 DM. They questioned the coexistence of type 2 DM at the time of SCFE diagnosis before surgical treatment. In their retrospective study evaluating patient-reported outcomes as well as comorbidities with an average of 19-year follow-up period, Escott et al. (2015) reported DM in 8% of cases. Moreover, they noted an increase in the mean BMI of 10 during postoperative follow-up. The rate of obesity and obesity-related comorbidities was detected as higher than in the general population. In our study, 7 patients were detected as at risk of DM and 1 patient was diagnosed as obvious type 2 DM according to HbA1c results. However, in the control group only 2 patients were detected as at risk of DM according to IFG levels. There was a statistically significant difference between the 2 groups regarding the mean of HbA1c levels and abnormal HbA1c frequency. Additionally, in a multivariate logistic regression with confounding factors that may affect HbA1C value, HbA1c levels were found to be related only to the SCFE group. Other variables, especially weight, which may have the potential to raise HbA1c levels were irrelevant. In the SCFE group, the frequency of patients with abnormal serum glucose level was higher than the ratio reported by Escott et al. (2015). They evaluated the coexistence of DM with SCFE preoperatively in patients who underwent surgical treatment. Additionally, we evaluated DM postoperatively at 3-year follow-up compared with obese patients without SCFE. Therefore, we recommend that the patients diagnosed with and treated for SCFE should be followed up for potential abnormalities of glucose metabolism, which may not be detected on the first admission.

Vitamin D deficiency has been noted in young patients admitted to hospital with orthopedic problems, as well as in healthy adolescents (Davies et al. 2011). Buyukinan and Ozen (2012) reported vitamin D deficiency in obese adolescents as high as 96%. According to a study analyzing the results after in situ pinning, a negative correlation was found between vitamin D levels and the fusion time in the physis line (Judd et al. 2015). Furthermore, Judd et al. (2015) also reported that only 2 of the 27 patients in their series had a normal serum vitamin D level at the time of SCFE diagnosis. On the other hand, Arkader et al. (2015) reported that all patients diagnosed with SCFE between the ages of 9 and 14 years had normal vitamin D levels. In their series of 15 cases with SCFE, Madhuri et al. (2013) also reported that none of the patients had normal serum vitamin D according to the tested reference range. None of our patients and controls had normal levels of vitamin D. Our patients and healthy individuals belong to the same ethnicity and live in the same city, and blood-taking periods were evenly distributed in all seasons of the year. Maybe surgeons dealing with the treatment of such patients should consider routine follow-up of vitamin D levels at least during the fusion time of the epiphysis after surgical intervention.

Although a possible relationship of SCFE with endocrinological disorders has been evaluated by several studies, routine clinical screening for endocrine pathologies has not been recommended due to low rate of reported coexistence (Ogden and Southwick 1977, Mann et al. 1988). We also noted normal serum levels of F-T3, F-T4, TSH, PTH, ALT, AST, creatinine, and urea in our patients.

We note some limitations of our study. First, it was a retrospective evaluation of a prospectively followed patient group. However, our cohort was a relatively large series of patients with a rare diagnosis. Furthermore, the main strength of the study was that we studied serum levels of many different parameters compared with a control group.

In summary, a risk of type 2 DM with abnormal HbA1c levels was significantly higher in patients with SCFE when compared with obese adolescents without SCFE. Additionally, an abnormal serum lipid profile and vitamin D insufficiency was similar in patients with SCFE when compared with obese/overweight adolescents.

HU, MD was involved in study design, manuscript preparation, manuscript writing, and is the corresponding author. YC, MD and EU, MSc were involved in data collection, statistical analysis, and manuscript editing. SD, MD carried out data collection and analysis of literature. HS, MD was involved in language editing and performed measurements. AIB, MD was the senior author, and involved in last revision of the manuscript.

*Acta* thanks anonymous reviewers for help with peer review of this study.
